# Occurrence and antibiogram of multidrug-resistant *Salmonella enterica* isolated from dairy products in Libya

**DOI:** 10.14202/vetworld.2022.1185-1190

**Published:** 2022-05-17

**Authors:** Aboubaker M. Garbaj, Tahani B. Ben Gawella, Jihan A. Sherif, Hesham T. Naas, Hanan L. Eshamah, Salah M. Azwai, Fatim T. Gammoudi, Said K. Abolghait, Ashraf A. Moawad, Ilaria Barbieri, Ibrahim M. Eldaghayes

**Affiliations:** 1 Department of Food Hygiene and Control, Faculty of Veterinary Medicine, University of Tripoli, Tripoli, Libya; 2 Department of Microbiology and Parasitology, Faculty of Veterinary Medicine, University of Tripoli, Tripoli, Libya; 3 Department of Food Hygiene and Control, Faculty of Veterinary Medicine, Suez Canal University, Ismailia 41522, Egypt; 4 Department of Food Hygiene and Control, Faculty of Veterinary Medicine, Cairo University, 12211 Giza 12211, Egypt; 5 Department of Genetics, The Lombardy and Emilia Romagna Experimental Zootechnic Institute, Via Bianchi 9, Brescia 25124, Italy

**Keywords:** 16S rDNA partial sequence, antibiotic resistance, dairy products, Libya, milk, *Salmonella* spp

## Abstract

**Background and Aim:**

Foodborne illnesses are a serious challenge to human health and the economic sector. For example, salmonellosis remains a burden in developed and developing nations. Rapid and reliable molecular methods to identify *Salmonella* strains are essential for minimizing human infection. This study aimed to identify *Salmonella* spp. in raw milk and dairy products using conventional and molecular techniques and to test the antibiotic susceptibility of the isolated strains.

**Materials and Methods:**

One hundred and thirty-one milk and dairy product samples were randomly collected from different localities in Libya. Samples were examined for the presence of *Salmonella* by conventional culture techniques, including cultivation in Rappaport-Vassiliadis broth and streaking on xylose lysine deoxycholate agar. Identification also used polymerase chain reaction and partial sequencing of 16S rDNA. Twenty-four antibiotics were used for the examination of antimicrobial resistance of *Salmonella* spp. isolates with the agar disk diffusion method (Kirby–Bauer technique). Multi-antibiotic resistance index and antibiotic resistance index (ARI)for *Salmonella enterica* isolates were calculated.

**Results:**

Twenty-one of 131 samples (16%) were positive for *Salmonella* spp. recovered from 9 (16%), 2 (11%), 4 (22.2%), and 6 (46%) samples of raw cow milk, fermented raw milk, and fresh locally made soft cheeses, Maasora and Ricotta), respectively. Samples of ice cream, milk powder, and infant formula showed no *Salmonella* spp. contamination. Only 9 of 21 (42.8%) isolates were confirmed as *S*. *enterica* by partial sequence 16S rDNA analysis. All isolates were resistant to amoxycillin, bacitracin, penicillin G, lincomycin, vancomycin, clindamycin, and cloxacillin with an ARI of 0.042. In contrast, all tested strains were sensitive to levofloxacin, doxycycline, and ciprofloxacin. In addition, all of the tested isolates (100%) were resistant to more than one antibiotic.

**Conclusion:**

This study demonstrated the applicability of molecular techniques, compared with conventional methods, as preferable for the identification of *Salmonella* in milk and dairy products and thus reduction of milk-borne transmission to the consumers. From the view of public health, isolation and identification of *Salmonella* multidrug-resistant strains from raw cow`s milk and locally prepared dairy products sold in the Libyan markets indicate the need to improve the handling and processing of milk and dairy products to minimize the prevalence of *Salmonella*, one of the most important foodborne microorganisms that cause food poisoning.

## Introduction

Milk and dairy products are basic components of healthy human diets. These products have a high nutritional value and several necessary elements that make them a complete food. Consequently, dairy products are susceptible to microbial contamination and are a favorable medium for the growth of various microorganisms, including pathogenic bacteria [1].

Raw milk and its products may become contaminated either directly or indirectly from different sources [2]. Pathogens in milk are a leading cause of foodborne infections, which is a collective public health issue for investigators and consumers [3]. Salmonellosis remains the major human foodborne illness transmitted through the consumption of contaminated milk and its products. This pathogen has been reported in different foods, including eggs, meat, vegetables, fruit juices, and dairy products [4,5]. *Salmonella* may originate from fecal contamination. The genus is a common intestinal tract group; hence, cross-contamination can occur at numerous points during the manufacture of dairy foods [6].

Recent investigation indicated that consuming contaminated eggs, meat, or milk with *Salmonella* are responsible for more than 90% of human salmonellosis [7]. Pasteurized milk can also be associated with some outbreaks [8].

Antibiotic-resistant pathogens have caused universal concern since the prevalence of multidrug-resistant (MDR) *Salmonella* increased. *Salmonella*, mainly MDR strains, were isolated from one dairy farm during salmonellosis eruptions where the same strains were detected in infected people [9]. The existence and spread of possibly more pathogenic stains could be obscured if identification is not fast and reliable. This issue could delay recovery of infected people and increase the risk of mortality [10].

Foodborne illness outbreaks are reported among Libyan consumers, which may require their hospitalization. However, isolation of causative agents is rare, and it is not possible to link foodborne pathogens in food samples with food poisoning. Researchers in Libya reported the isolation and molecular identification of *Escherichia coli* O157, *Cronobacter sakazakii*, and *Cronobacter pulveris* from milk and other dairy products [11,12]; however, no attempts are reported to identify *Salmonella* *enterica*. Antibiotic resistance patterns of *Salmonella* are highly variable for the most common antibiotics [13].

There is a high demand for accurate and rapid identification of pathogens in many countries, such as Libya, where consumption of unpasteurized dairy products is not uncommon. The application of traditional culture methods has irreducible limitations and may take 4-7 days to provide provisional identification of *Salmonella* [7]. This study aimed to identify *Salmonella* spp. in raw milk and dairy products from different localities in Libya using conventional and molecular techniques and to test the isolated strains against a variety of antibiotics to determine their susceptibility.

## Materials and Methods

### Ethical approval

No ethical approval was required for this study. However, samples were collected as per standard sample collection methods.

### Study period and location

Samples of milk and dairy products were collected from different Libyan cities (Tripoli, Sabha, Tobruk, and Regdalin) (Table-1) from January 2019 to December 2020.

**Table 1:** Incidence of *Salmonella* spp. in milk and dairy products samples.

Type of samples	Number of samples	Number of positive samples	Incidence %
Raw cow’s milk	56	9	16
Fermented milk	18	2	11
Milk powder	18	None	None
Maasora cheese	13	4	22
Ricotta cheese	13	6	46
Ice cream	13	None	None
Total	131	21	16

### Sample collection and preparation

A total of 131 samples were collected aseptically and transported as quickly as possible to the Laboratory of Food Hygiene and Control Department at the Faculty of Veterinary Medicine, University of Tripoli. Samples were assessed by conventional and molecular methods for the identification of *Salmonella* spp. and examined for antimicrobial susceptibility. Samples were prepared following the protocol of the American Public Health Association [14].

### Isolation and purification

One milliliter of sample homogenate was aseptically transferred to 9 mL of sterile trypticase soya broth supplemented with yeast extract (CM 131, Oxoid, Hampshire, UK). Inoculated tubes were incubated at 43°C for 24 h. A loopful from each broth culture was inoculated into 10 mL of Rappaport-Vassiliadis (RV) broth (RV, 61075, Liofilchem, Italy) and incubated at 42±0.2°C for 24±2 h. Enrichment RV broth was thoroughly mixed, and a loopful was streaked onto bismuth sulfite (BS) agar (M 4027, HiMedia, India) and xylose lysine deoxycholate (XLD) agar (CM 0469, Oxoid, Hampshire, UK) plates and incubated at 35°C for 24±2 h. Suspect brown, gray, or black colonies with or without a metallic sheen on BS agar and pink colonies with or without black centers on XLD agar were picked and recultured on nutrient agar (Park Scientific, Northampton, UK) slants for further identification.

### Biochemical identification

Suspect isolates were identified biochemically using a KBO HiSalmonella identification kit (HiMedia). Each kit contains a combination of 12 tests (MR, VP, urease, H_2_S, citrate, and lysine utilization, *Ortho*-Nitrophenyl-β-galactoside, and five different carbohydrates). Procedures followed the manufacturer’s instructions.

### Molecular identification of *Salmonella* spp.

Genomic DNA extraction of biochemically identified *Salmonella* spp. used a single colony from XLD citrate agar plates with a GF-1 bacterial DNA extraction kit (#GF-BA-100, Vivantis, Malaysia). Procedures followed the manufacturer’s protocol as previously described [15]. Partial 16S rDNA was amplified using universal oligonucleotide primers [16].

### Antimicrobial susceptibility testing

*S. enterica* strains were stored at 4°C on nutrient agar plates (Park Scientific). *Salmonella* isolates were propagated from single colonies inoculated into 5 mL of brain heart infusion broth (BH, 610008, Liofilchem, Italy) and incubated for 18 h at 37±2°C. Twenty-four antibiotic disks (Oxoid, England) were selected and used for the antimicrobial resistance of *Salmonella* spp. isolates using agar disk diffusion (Kirby–Bauer technique) and as recommended by the Clinical and Laboratory Standards Institute [17]. Multi-antibiotic resistance (MAR) index and antibiotic resistance index (ARI) for *S. enterica* isolates were calculated as previously described [18].

## Results

**Figure-1:**
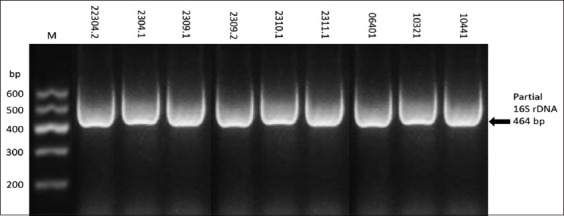
Representative gel of partial identification of 16S rDNA (464 bp) products of isolated *Salmonella enterica*.

Only 21 of 131 cultured samples (16%) yielded bacterial growth on XLD that suggested *Salmonella* spp. suspect colonies appeared red or yellow with black centers. *Salmonella* was recovered from 9 of 56 (16%) raw cow’s milk samples; no *Salmonella* spp. were identified in ice cream or milk powder samples; *Salmonella* was identified in 4/18 (22.2%) of Maasora cheese, 6 of 13 (46.1%) of Ricotta cheese, and 2 of 18 (11.1%) fermented milk samples (Table-1). Cheese thus showed the highest incidence and fermented mile the lowest. Biochemical tests of suspect isolates used the KBO HiSalmonella identification kit. Bacteria were incubated at 37°C for 18-24 h, reagents were added, and reactions developed. Color changes were recorded. Extracted DNA was amplified using polymerase chain reaction (PCR) amplification with partial 16S rDNA primers. Isolates were assessed by gel electrophoresis. Molecular analysis identified only nine of 21 isolates as *S*. *enterica* (Table-2). Seven isolates were recovered from raw cow’s milk and one each from Maasora and Ricotta cheese (Table-3 and Figure-1).

**Table 2:** Comparison between the results of XLD medium and 16S rDNA technique for the identification of *Salmonella* spp.

Type of samples	Number of samples	Number of positive samples	

		Selective media (XLD) (%)	16S rDNA (%)
Raw cow’s milk	56	9 (16)	7 (77)
Fermented milk	18	2 (11)	None
Milk powder	18	None	None
Maasora cheese	18	4 (22)	1 (25)
Ricotta cheese	13	6 (46)	1 (16)
Ice cream	13	None	None
Total	131	21 (16)	9 (42.8)

XLD=Xylose lysine deoxycholate

**Table 3:** Identification of suspected *Salmonella* spp. by PCR and partial sequencing of 16S rDNA.

Blast NCBI search results	Homology %	Isolate code	Type of sample
*Salmonella enterica* subsp*. enterica*	100	2304.2	Raw cow’s milk
*Salmonella enterica* subsp*. enterica*	100	2304.1	Raw cow’s milk
*Salmonella enterica* subsp*. enterica*	100	2309.1	Raw cow’s milk
*Salmonella enterica* subsp*. enterica*	100	2309.2	Raw cow’s milk
*Salmonella enterica* subsp*. enterica*	100	2310.1	Raw cow’s milk
*Salmonella enterica* subsp*. enterica*	100	2311.1	Raw cow’s milk
*Salmonella enterica* subsp*. enterica*	100	1032.1	Raw cow’s milk
*Salmonella enterica* subsp*. enterica*	100	10441	Ricotta cheese
*Salmonella enterica* subsp*. enterica*	100	6401	Maasora cheese

PCR=Polymerase chain reaction

### Antibiotics resistance patterns

Isolated *S. enterica* were tested for susceptibility to antibiotics by disk diffusion on Mueller-Hinton agar plates [17]. Isolates showed varying inhibition zones surrounding 24 antibiotic disks. All isolates were resistant to amoxycillin, bacitracin, penicillin G, lincomycin, vancomycin, clindamycin, and cloxacillin with ARI of 0.042. Conversely, all strains were sensitive to levofloxacin, doxycycline, and ciprofloxacin. Strains showed different degrees of sensitivity to other antibiotics. In addition, all isolates (100%) were resistant to more than 1 antibiotic. Approximately 89% of the tested isolates were resistant to ≥11 drugs. Further, 56% of the tested isolates were resistant to ≥12 (50%) antibiotics. *S. enterica* isolates proved to be extremely drug-resistant (Table-4). MAR indices ranged from 0.42 to 0.67 (≥0.20 is significant) (Table-5).

**Table 4:** Antibiotic susceptibility profiles of *Salmonella enterica* isolated from milk and dairy products.

Antibiotic	S%	I%	R%	ARI
Amoxycillin	-	-	100	0.042
Amoxycillin/clavulanic acid	67	22	11	0.005
Ampicillin	89	-	11	0.005
Bacitracin	-	-	100	0.042
Penicillin G	-	-	100	0.042
Methicillin	-	11	89	0.037
Erythromycin	-	22	78	0.032
Gentamicin	78	22	-	0.000
Kanamycin	44	56	-	0.000
Lincomycin	-	-	100	0.042
Tobramycin	56	44	-	0.000
Vancomycin	-	-	100	0.042
Levofloxacin	100	-	-	0.000
Clindamycin	-	-	100	0.042
Cefotaxime	67	33	-	0.000
Doxycycline	100	-	-	0.000
Ciprofloxacin	100	-	-	0.000
Cloxacillin	-	-	100	0.042
Nitrofurantoin	89	-	11	0.005
Oxytetracycline	89	-	11	0.005
Streptomycin	-	100	-	0.000
Tetracycline	89	-	11	0.000
Chloramphenicol	89	-	11	0.005
Sulfamethoxazole/trimethoprim	89	-	11	0.005

ARI=Antibiotic-resistant index.

**Table 5:** MAR index of *Salmonella enterica* isolated from milk and dairy products.

S. No.	Isolate code	MAR index
1	2304.2	0.46
2	2304.1	0.46
3	2309.1	0.50
4	2309.2	0.46
5	2310.1	0.54
6	2311.1	0.67
7	6401	0.50
8	1032.1	0.54
9	10,441	0.42

MAR=Multi-antibiotic resistance

## Discussion

Raw milk has high nutritional content and provides a good medium for growth and multiplication of microorganisms, including *Salmonella*. These microorganisms may contaminate milk in various ways, which may lead to different results. Some microorganisms can facilitate fermentation, and others may cause spoilage. Further, some microorganisms promote health and others cause disease [19]. Isolation and identification of microorganisms using traditional methods are time-consuming. Rapid detection of foodborne microorganisms, such as *Salmonella*, will facilitate the prevention of disease by these pathogens [20]. The present study on *Salmonella* spp. evaluated 131 samples of different milk and other dairy products collected from different locations in Libya. The incidence of *Salmonella* spp. was 16%, with the highest incidence recorded in samples of Ricotta cheese (46.1%). This finding might be explained by contamination during preparation and processing or contamination of equipment and utensils. Alternatively, milk in the bulk tank can be contaminated in the udder or through fecal matter.

*Salmonella* spp. are a leading cause of foodborne disease, and consumption of milk has been implicated in salmonellosis outbreaks. Strains of *Salmonella* resistant to multiple antibiotics have been isolated from dairy cows during such outbreaks in dairy operations [21]. The incidence of *Salmonella* in raw cow’s milk was 16%, yet other studies did not report *Salmonella* in this product [22,23]. Still, the consumption of raw milk is recognized as a common means of transmission of *Salmonellae* to people. This transmission could, as mentioned, be related to the direct contamination of raw milk during milking or through milking equipment and bulk storage tanks [24]. Awareness of hazards associated with the consumption of unpasteurized milk is needed among consumers [25]. Moreover, at least six outbreaks of *Salmonella* infection in infants were related to the consumption of contaminated powdered infant formula [26].

In the present study, the incidence of *Salmonella* in Maasora cheese was 22.2%, and in Ricotta, 46.1%. Ricotta cheese has a higher water content than Maasora, which might be more favorable for bacterial growth. Conversely, Esho *et al*. [27] did not isolate *Salmonella* from natural cheese. Our findings seem consistent with Torres-Vitela *et al*. [28], who examined 200 samples of two types of fresh cheese (Panela and Adobera) for *Salmonella* spp. and found an incidence of 56% in Panela and 20% in Adobera samples. *Salmonella* spp. can be a contaminant in milk obtained from infected herds. An outbreak of *Salmonella muenster* was reported in France in 25 cases linked to the consumption of goat cheese made from raw milk [29]. Moreover, 85 cases of illness linked to the consumption of Cotija cheese made from raw milk were reported in the United States during 2006-2007 [30].

Ice cream is a frozen dairy dessert made from a variety of ingredients. *Salmonellae* do not survive typical minimum pasteurization processes. The presence of such microorganisms indicates that this process was improper or that contamination occurred after pasteurization. Therefore, sanitation is critical for ensuring that dairy products do not become recontaminated [31]. In our study, none of 13 samples of ice cream showed growth of *Salmonella* spp. In contrast, Baylis *et al*. [32] detected low levels of *Salmonella typhimurium* in ice cream. This variation is likely attributed to the use of various analytical techniques and sampling patterns. Differences might also be explained by the specific geographical distribution of genera [33]. Molecular confirmation of *Salmonella* spp. identified nine strains; these isolates showed typical characteristics of *Salmonella* spp. on XLD selective media. Isolates were first identified as *Salmonellae* by biochemical characteristics. These suspect isolates were then assessed using PCR and 16S rDNA partial sequencing. All nine isolates belonged to *S. enterica* subsp. *enterica*. The PCR assay can be used to identify *Salmonella* spp. The process is simple, specific, and can detect this species rapidly. Moreover, the application of PCR can resolve several difficulties with the use of traditional identification. The method can provide more reliable results than conventional methods. This conclusion is supported by Gwida and Al-Ashmawy [25], who identified *Salmonella* spp. using PCR in 21% (42/200) of milk and dairy products. In comparison, Esho *et al*. [27] did not isolate *Salmonella* spp. from any natural cheese samples using real-time PCR. Several techniques and many studies are used to identify *Salmonella* spp. in food samples, especially for unpasteurized milk and milk products. *Salmonella*e are important and common enteric pathogens that can be transmitted through the consumption of contaminated dairy products. *Salmonella* infection remains a significant public health concern in many countries where antibiotic-resistant strains are high.

### Antibiotics resistance patterns

The increased emergence of MDR bacteria is worrying and has become a critical issue worldwide [34]. Coordination among sectors under the One Health Concept is needed to understand the development of resistant pathogens in humans, animals, and the environment. The primary source of antibiotics in animal products is due to overuse or misuse (prophylactic or subtherapeutic administration) of antimicrobial agents in animal feed. These practices increased antimicrobial resistance among bacterial strains, especially in developing countries [35]. The present study showed that all tested strains have patterns of sensitivity and resistance levels to 24 antibiotics. These antibiotics are widely used in human and veterinary medicine. The majority of isolated *S. enterica* were resistant to amoxycillin, bacitracin, penicillin G, lincomycin, vancomycin, clindamycin, and cloxacillin with an ARI of 0.042.

## Conclusion

The results obtained in this study showed that 21 of 131 (16%) samples showed *Salmonella* spp. based on conventional methods of culturing in RV broth, XLD agar, and biochemical identification. Incidence was particularly high in soft cheeses, Maasora and Ricotta. However, based on partially sequenced 16S rDNA, PCR identified only nine isolates of *S. enterica* in raw cow’s milk and locally produced soft cheese. PCR methods have been widely used for the identification of *Salmonella* spp. worldwide; however, this work is the first molecular confirmation of *S. enterica* subsp. *enterica* in Libya. Using molecular techniques for identification of foodborne microorganisms is a preferred choice for rapid and accurate diagnosis, better detection and early treatment of illnesses. MAR indices of the tested isolates ranged from 0.42 to 0.67 (≥0.2 is considered high). Thus, isolates were highly resistant to a wide range of commonly used antibiotics.

## Authors’ Contributions

AMG, SMA, FTG, SKA, AAM, and IME: Designed and planned the study. TBG, AMG, JAS, HTN, HLE, SMA, FTG, SKA, AAM and IME: Collected the samples and did the laboratory work. IB: Sequencing of the PCR products in her laboratory (IZSLER Laboratory in Brescia, Italy). All authors contributed equally in the preparation and revision of the manuscript. All authors have read and approved the final manuscript.
